# Dual-responsive and Multi-functional Plasmonic Hydrogel Valves and Biomimetic Architectures Formed with Hydrogel and Gold Nanocolloids

**DOI:** 10.1038/srep34622

**Published:** 2016-10-05

**Authors:** Ji Eun Song, Eun Chul Cho

**Affiliations:** 1 Department of Chemical Engineering, Hanyang University, Seoul, 04763, South Korea

## Abstract

We present a straightforward approach with high moldability for producing dual-responsive and multi-functional plasmonic hydrogel valves and biomimetic architectures that reversibly change volumes and colors in response to temperature and ion variations. Heating of a mixture of hybrid colloids (gold nanoparticles assembled on a hydrogel colloid) and hydrogel colloids rapidly induces (within 30 min) the formation of hydrogel architectures resembling mold shapes (cylinder, fish, butterfly). The biomimetic fish and butterfly display reversible changes in volumes and colors with variations of temperature and ionic conditions in aqueous solutions. The cylindrical plasmonic valves installed in flow tubes rapidly control water flow rate in on-off manner by responding to these stimuli. They also report these changes in terms of their colors. Therefore, the approach presented here might be helpful in developing new class of biomimetic and flow control systems where liquid conditions should be visually notified (e.g., glucose or ion concentration changes).

In addition to drug delivery systems^1^ and tissue engineering^2^, responsive hydrogels have been used as sensors to detect various molecules^3^,^4^,^5^,^6^,^7^,^8^,^9^,^10^,^11^,^12^,^13^,^14^,^15^,^16^,^17^,^18^,^19^,^20^, valves to control flow rate of fluids^21^,^22^,^23^,^24^,^25^,^26^, and actuators for various bio-applications^27^,^28^,^29^,^30^,^31^. Hydrogel optical sensors are made by embedment of photonic crystals of polymer/inorganic nanoparticles in hydrogel matrices^4^,^6^,^7^,^8^,^9^,^10^,^11^,^12^,^13^,^14^,^15^,^16^,^17^,^18^,^19^,^20^. Optical changes (e.g., colors) of hydrogels are caused by the variation of the photonic crystal structures in compliance with the dimensional changes of hydrogel matrices upon notice of environmental changes or detection of various target molecules. Hydrogel valves are mostly made through photo-polymerization reactions of responsive polymer precursors. The valves are especially useful for the flow control of fluids in microfluidic channels because the relaxation (response) times of gels are becoming shorter with decreasing the sizes of hydrogels: the times are generally proportional to the square of the gel sizes^32^,^33^. Hydrogel actuators are made with various shapes sufficiently to exhibit the desired properties^27^,^28^,^29^,^30^,^31^,^34^.

To date, the responsive hydrogels as a valve or actuator have mostly been used for only one purpose. However, addition of sensing functions to the valves or actuators might bring a synergistic effect, as in the case of theranostic (therapeutic + diagnostic) system, thereby expanding their applications. Construction of such architectures might also provide a fundamental basis for the fabrication of biomimetic hydrogel systems: some sea animals (fish and squid) and butterflies alter their internal structures (or shapes) and, hence, colors according to environmental changes^35^,^36^. However, the majority of hydrogels used as optical sensors have been prepared on substrates with a μm-scale thickness, which is insufficient to produce bulk-scale three-dimensional structures. Regarding this issue, Hu and co-workers prepared visual-scale objects in which well-ordered stimuli-responsive hydrogel colloids were chemically linked^37^,^38^ while investigating the photonic crystal behaviours of various hydrogel colloids^37^,^38^,^39^,^40^,^41^,^42^,^43^. With a perspective of response time, the approach would provide additional merit because the responses of building block colloids influence the response rate of colloidal hydrogel architectures^44^. Therefore, the fast response of valves or actuators might be anticipated despite the construction of large-sized hydrogel structures. However, process conditions for the bulk-scale photonic crystal structures were too strict and it took too long time to produce various/desired shapes under various conditions. Moreover, the processability (moldability) for production of colloidal-based hydrogel architectures has not been fully demonstrated. That is, the structures must be manufactured with any shape by resembling the mold’s geometry. Thus, it is still a challenge to find a new strategy adding the optical sensing functions to hydrogel valves and actuators for versatile applications in biomedical fields.

To address these issues, here we present a straightforward approach for three-dimensional plasmonic hydrogel valves and architectures that respond to both temperature and ions (dual-responsive) by means of volume and color change (multi-functional) in aqueous systems (Fig. 1a). The hydrogel architectures are spontaneously formed by heating an aqueous mixture of hydrogel colloids and hybrid colloids where gold nanoparticles (Au NPs) are assembled on a hydrogel colloid. These two colloids have thermo- and ionic-sensitive functions, and thus the architectures respond to these stimuli. Color changes of the architecture are attributable to the hybrid colloids that vary optical characteristics (due to the variation of assembled structure of Au NPs) according to these stimuli. The moldability is proven by creating various architectures (cylinder, butterfly and fish) that closely resemble the shape of molds. We use the architectures as plasmonic valves which rapidly and reversibly control aqueous fluid flow rates in on-off manner by responding to changes in temperature and ions (types, concentration) of the water. At the same time, the valves also report these changes by their colors.

## Results and Discussion

The formation of three-dimensional hydrogels structures based on the assembly of hydrogel colloids have been studied by Saunders *et al*.^47^,^48^,^49^,^50^,^51^,^52^,^53^, Weitz *et al*.^44^,^54^, and other scientists^55^. They explored ways to enhance functions of the structures. Examples included the demonstration of temperature- and pH-responsibility, fast response to stimuli, fast formation of the structures, the increase in their mechanical properties through the engineering chemical structures of colloids, and the possibility of composite hydrogel structures bearing various functional materials (proteins, drugs, magnetic nanoparticles). However, the applicability of this approach has not fully demonstrated to date. As mentioned in the introduction, the formation of colloidal structures bearing bulk-scale photonic crystal characters suggested their possibilities as optical sensors^37^,^38^, but the strict process conditions may limit the production of large-scale three-dimensional architectures having various shapes.

### Synthesis and characterization of hydrogel colloids and hybrid colloids

**Figure 1:**
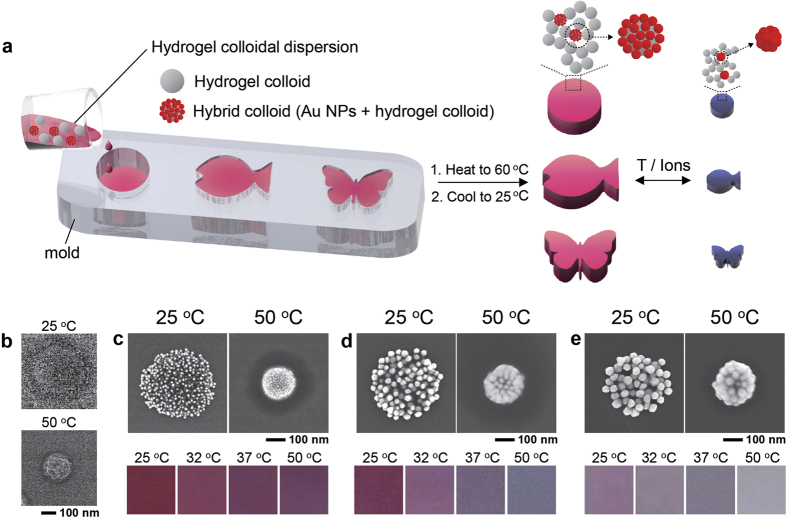
Fabrication of colloidal plasmonic hydrogel architectures consisting of hydrogel colloids and hybrid colloids. (**a**) A schematic illustrating the fabrication process of colloidal plasmonic hydrogel architectures and their responses to temperature and ionic changes. (**b**) Scanning electron microscopy images showing the temperature-dependent changes in size of the hydrogel colloid used in the present study. (**c–e**) Top: scanning electron microscopy images showing the temperature-dependent morphologies of hybrid nanocolloids formed with a hydrogel colloid and Au NPs having (**c**) 15 nm, (**d**) 33 nm, and (**e**) 50 nm. Bottom: colors of the hybrid colloids in deionized water at various temperatures.

Our first objective in this research was to develop colloidal-based, stimuli-sensitive hydrogel architectures that express their responses via volume and color changes. When developing a method, we bore in mind that the architectures should be easily and rapidly produced for practical uses. In this regard, we introduced our previous reports to rapidly produce architecture through a simple heating of aqueous dispersion containing hydrogel colloids^44^,^54^. Poly(*N*-isopropylacrylamide-co-allyamine), p(NiPAAm-co-AA), hydrogel colloids (Fig. 1b) were synthesized via suspension polymerization. The hydrodynamic diameters and zetapotentials of the colloids were 476 nm and +17 mV at 25 °C and 248 nm and +31 mV at 50 °C. During heating to above transition temperature of the colloids (>35 °C), the as-prepared hydrogel colloidal dispersion formed clusters and eventually three-dimensional networks. During the synthesis of hydrogel colloids, some water-soluble p(NiPAAm-co-AA) chains might be also synthesized either with chemical compositions different from the p(NiPAAm-co-AA) hydrogel colloids or with their chain lengths being short enough for solubilization in water. We speculated these polymers could play a key role to construct the hydrogel architectures because the architectures cannot be formed after the removal of aqueous solution and the colloids are re-dispersed in deionized water. As such, both depletion force and hydrophobic interaction between the hydrogel colloids could be involved for the colloidal assembly while heating the as-prepared hydrogel colloid dispersion above transition temperature^54^. However, the colloids randomly arranged in the hydrogel architectures, and thus they lacked in photonic crystal characteristics for optical changes with stimuli. We supplemented this function by introducing a small amount of hybrid colloids with Au NPs assembled on the hydrogel colloids, which were identical to those used as building blocks for the architectures. Three types of hybrid colloids respectively bearing spherical Au NPs of 15, 33, and 50 nm in diameter (Fig. 1c–e) were prepared by simply heating mixtures of Au NPs and the hydrogel colloids to 50 °C, due to the electrostatic attraction and hydrophobic forces between the colloid and Au NPs^46^. The Au NPs on the hydrogel colloids reversibly altered their assembly structures according to temperature changes in deionized water (Fig. 1c–e), exhibiting reversible color changes. The number of variable colors was dependent on the sizes of the spherical Au NPs. The changeable spectral bandwidth generally increased with increasing sizes (Supplementary Fig. S1). It is important to note that the use of identical hydrogel colloids both for the hybrid colloids and for the hydrogel architectures resulted in the same responsiveness of the two nanocolloids, as well as architectures, to stimuli (Supplementary Fig. S2). Thus, the volume and color changes of the hydrogel architectures could occur simultaneously.

### Preparation of cylindrical plasmonic hydrogel architectures and temperature-dependent volume and color changes of the architectures

**Figure 2:**
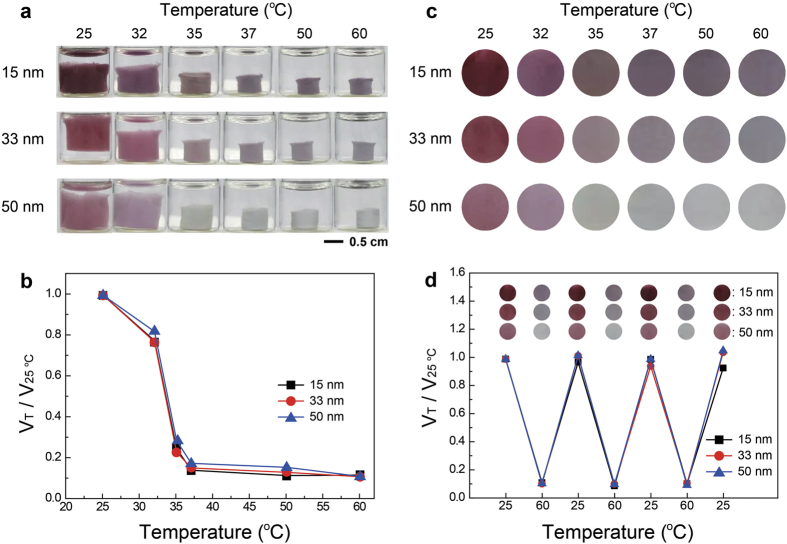
Temperature-induced volumetric and color changes of colloidal plasmonic hydrogel architectures. (**a**) Photographs showing temperature-dependent changes in the shape and color of colloidal plasmonic hydrogel architectures. Three types of hybrid colloids bearing Au NPs 15, 33, and 50 nm in size were used for the architectures. (**b,c**) Temperature-dependent changes in (**b**) volume and (**c**) color of the three architectures. (d) Volume and color changes of the three plasmonic architectures under repeated heating (60 °C) and cooling (25 °C) cycles.

The hybrid colloids were homogeneously mixed with the hydrogel colloids in an aqueous system: the number ratios of hybrid to hydrogel colloids were 1:107, 1:276, 1:524 when using the hybrid colloids bearing 15, 33, and 50 nm Au NPs, respectively. While the colloidal aqueous mixtures were heated in cylindrical glass vials to 60 °C, the architectures were formed within 30 min (Fig. 2a and Supplementary Fig. S3). Then, the clear aqueous solution was removed, deionized water at 25 °C was added to the hydrogel architecture, and the vial was heated again to 60 °C. The washing process was repeated one more time. The volume and color changes of three cylindrical hydrogels were investigated as shown in Fig. 2a. The three hydrogels rapidly decreased in volume between 32 and 37 °C, which is the transition temperature of hydrogel colloids^54^. The dimensional changes were identical for the three hydrogels (Fig. 2b), but the color changes differed depending on the size of Au NPs in the hybrid colloids (Fig. 2c). Wine to violet transition was observed in 15-nm Au NPs, and various colors were displayed by 33-nm Au NPs, implying that the highest temperature sensitivities were achieved with the hybrid colloids. The hydrogels with 50-nm Au NPs also displayed at least three colors. The three hydrogels had fairly good reversibility in their volumes and colors under repeated heating-cooling cycles (Fig. 2d). It is also worth noting that simple addition of Au NPs (not hybrid colloids) to the hydrogel colloids did not cause appreciable color changes of the architectures (Supplementary Fig. S4), indicating that significant color changes were achievable only when the arrangement of the Au NPs was well regulated.

Response time of hydrogel architectures containing hybrid colloids (with 15-nm Au NPs) were investigated (Supplementary Fig. S5). The response times (relaxation times) were found to be 67–75 s and the values were not much different from that of hydrogel architectures without hybrid colloids (82 s). Moreover, the relaxation times were more than 70 times shorter than that of bulk hydrogels (5.9 × 10^3^ s) of the same chemical compositions and equivalent sizes, implying the hydrogel architectures can rapidly respond to stimuli.

### Ion-dependent volume and color changes of plasmonic hydrogel architectures

**Figure 3:**
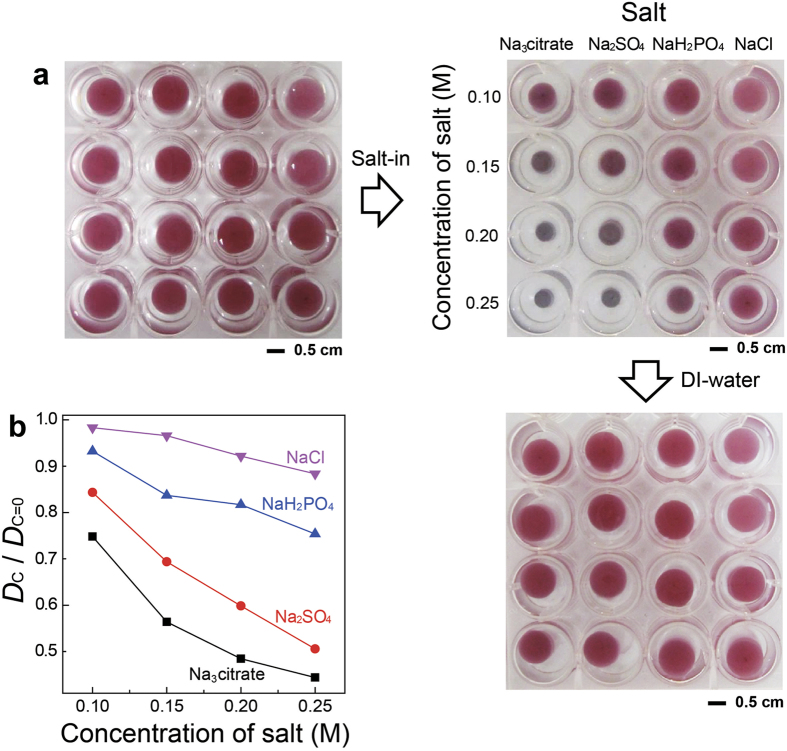
Ion-induced volumetric and color changes of colloidal plasmonic hydrogel architectures. (**a**) Photographs showing ion-dependent changes in the shape and color of colloidal plasmonic hydrogel architectures (cylindrical shape). The architectures were made with hydrogel and hybrid colloids bearing 33-nm Au NPs. (**b**) Changes in diameter (*D*) of the plasmonic hydrogel architectures with variation of salt types and concentrations. *D*_c=0_ and *D*_c_ indicate the diameter of the hydrogel in deionized water (*D*_c=0_) and in salt-containing aqueous solution at a certain concentration (*D*_c_).

We found that the hydrogels also responded to ions (Fig. 3). The cylindrical hydrogels, formed with hydrogel and hybrid nanocolloids bearing 33-nm Au NPs, had a pink-red/wine color in deionized water. Their colors and dimensions changed when deionized water was replaced with aqueous salt solutions (Fig. 3a). Significant volume and color changes (to dark blue) were observed with the addition of Na_3_citrate; Na_2_SO_4_ and NaH_2_PO_4_ induced moderate changes of the hydrogels; and no significant changes were observed with NaCl. Figure 3b shows the changes in the diameter of the hydrogel architectures. It was reported that transition temperatures of thermo-sensitive polymers decrease with the following salt power: Citrate^3−^ > CO_3_^2−^ > SO_4_^2−^ > H_2_PO_4_^−^ > F^−^ > Cl^−^ > Br^−^ > NO^3−^ > I^−^56, which is consistent with the present results. After replacing the salt-containing aqueous solutions with deionized water, all the hydrogels returned to their original volume and color, demonstrating reversibility.

### Fabrication of biomimetic plasmonic hydrogel architectures and temperature- and ion-dependencies test of the architectures

**Figure 4:**
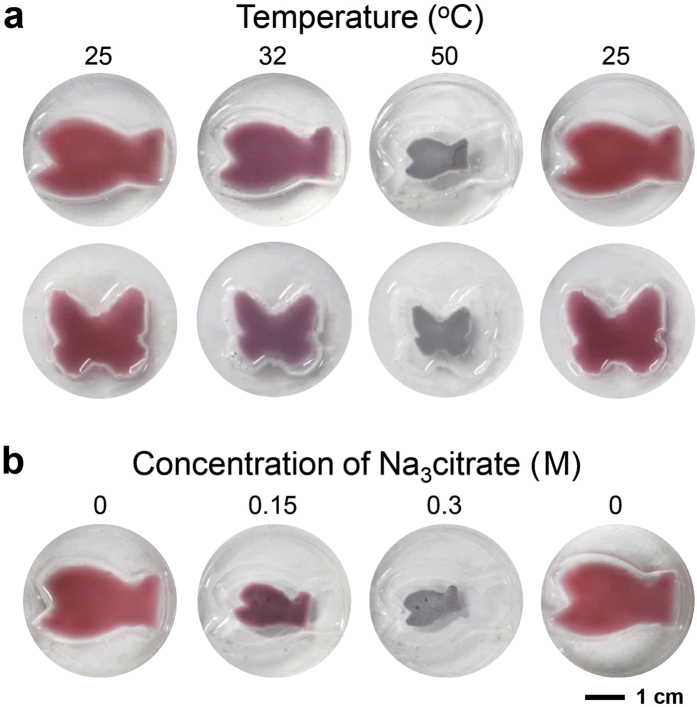
Moldability of plasmonic hydrogel resembling the shapes of molds. (**a**) Photographs showing temperature-dependent changes in the shape and color of plasmonic hydrogel fish (top) and butterfly (bottom). (**b**) Photographs showing changes in the shape and color of plasmonic hydrogel fish after immersion in deionized water and Na_3_citrate aqueous solution.

From Fig. 2, the hydrogels formed in the cylinder vial have a cylindrical shape, implying that the shapes of the hydrogels might resemble those of the molds. We demonstrated this by making two poly (dimethylsiloxane) (PDMS) molds with fish and butterfly shapes to produce biomimetic architectures which alter their internal structures (or shapes) and, hence, colors according to environmental changes^35^,^36^. The aqueous dispersion containing the hydrogel and hybrid colloids (containing 33-nm Au NPs) were poured into the molds and heated to 60 °C for 30 min. The washing step was as described above. Fig. 4a is photographs of plasmonic hydrogel fish and butterfly at various temperatures in deionized water. The sizes of the biomimetic architectures decreased with temperature increases, and their colors changed from pink red/wine (25 °C) to violet at 32 °C and to dark blue at 50 °C. When the temperature was decreased to 25 °C again, the fish and butterfly became bigger and turned pink red/wine again. The plasmonic hydrogel fish also changed in size and colors with a variation of the concentration of Na_3_citrate (Fig. 4b). Reversibility was also confirmed after replacing the salt-containing aqueous solution with deionized water. These results clearly demonstrate that various geometries of plasmonic hydrogel resembling the shapes of molds can be easily manufactured without losing their functions.

### Fabrication and flow rate “on-off tests of dual-responsive plasmonic hydrogel valves

**Figure 5:**
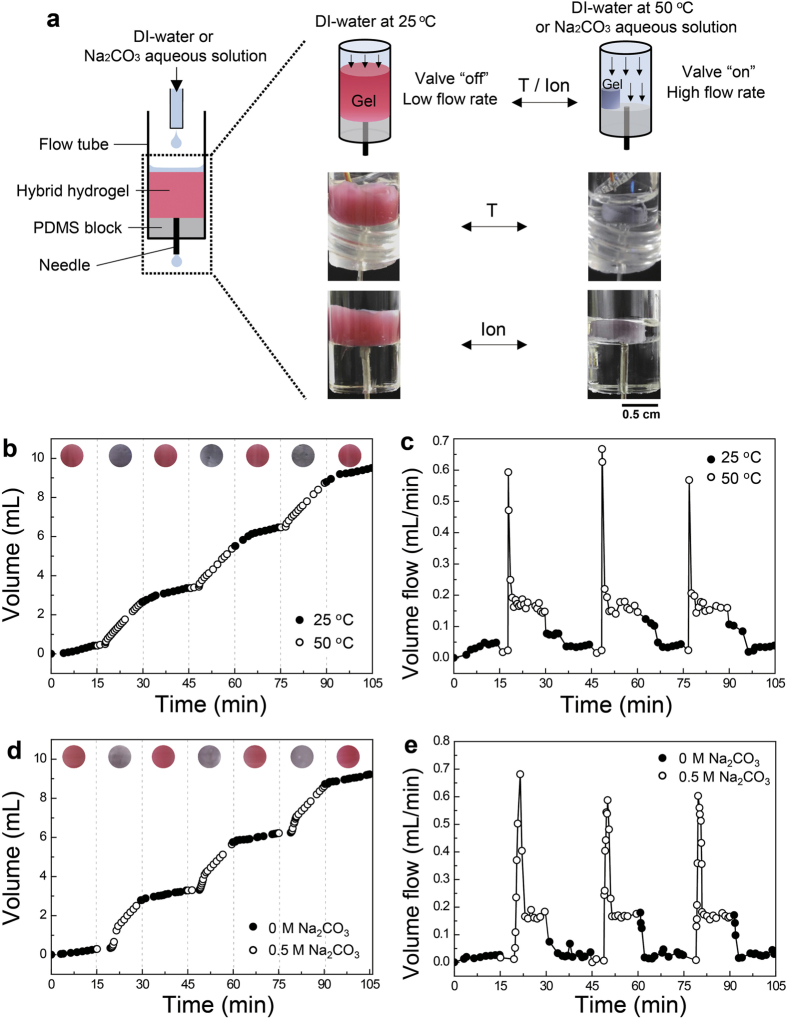
Dual-responsive valves that control the flow rate of aqueous fluids in an “on-off” manner. (**a**) A schematic illustrating a flow control system containing a colloidal plasmonic hydrogel valve both to control the flow rate of deionized water or salt-containing aqueous in response to temperature or ions of water. The valve was opened when either temperature or ionic concentration was increased, increasing the flow rate of fluid. The colors of valves changed according to these stimuli. (**b**) Cumulative volume and (**c**) flow rate of water in the outlet (needle) when the temperature of water at 25 and 50 °C was repeatedly switched every 15 min. (**d**) Cumulative volume and (**e**) flow rate of aqueous fluids in the outlet when deionized water and Na_2_CO_3_ aqueous solution (0.5 M) were alternatively run into the flowing tube every 15 min. Inset in (**b,d**): the colors of valve (**b**) at each water temperature or (**d**) when deionized water and Na_2_CO_3_ aqueous solution ran into the tube.

For a practical application, we designed dual-responsive valves that control the flow rate of aqueous fluids. Figure 5a shows a schematic illustrating the system. The plasmonic valve (with 33-nm Au NPs) was fabricated in a cylindrical glass vial to fit the shape of the flow system. The hydrogel was inserted into a flow tube where either deionized water or salt-containing aqueous solution entered from a syringe pump and went out through a needle connected to a PDMS block in the tube. The temperature of the flow tube was modulated by using a water circulating tube surrounding the outside of the flow tube. The flow rate of aqueous solution was checked every 30 s by measuring the cumulative weight of the aqueous solution going out of the flow system. Figure 5b is the cumulative volume of deionized water passing through the flow tube during repeated heating (to 50 °C) and cooling (to 25 °C) of the flowing water every 15 min. The cumulative amount of water at 50 °C passing through the flow tube was higher than that of water at 25 °C, because the plasmonic valve was opened (“on” state due to the volume decrease) at 50 °C and closed (“off” state due to the volume increase) at 25 °C. The valve also indicated “on” and “off” states by switching a violet/dark blue and pink red/wine color, respectively. Figure 5c shows the flow rate of hot and cold water passing through the plasmonic valve. The average water flow rate was 0.035 mL/min at 25 °C. The flow rates rapidly increased within 1.25 min (similar to the large influx of tap water when faucets are opened) after the circulating tube containing hot water (50 °C) was switched, indicating a fast valve response. At a steady state, the mean flow rate was 0.16 mL/min. The valves again rapidly decreased the flow rate and reached another steady state within 3.6 min after the circulating tube at 25 °C was switched.

The plasmonic valve also modulated the flow rates of aqueous fluids in response to a salt-containing aqueous solution. As shown in Fig. 5d and e, the flow rate increased when an aqueous Na_2_CO_3_ solution (0.5 M) flowed through the system because the valve was “on” state due to the decrease in hydrogel volume. The color changed from pink red/wine (off) to violet/dark blue (on). The valve reached the “on” state 4.6 min after the flow of salt-containing water, whereas it took 1.9 min to steadily reach the “off” state after the flow of deionized water. These results demonstrate that the plasmonic hydrogel architectures were effective for dual-responsive flow valves with reversible signal color displays for on/off states.

It is worth mentioning the two issues for practical uses of the present system. First, mechanical properties of the hydrogel architectures should be guaranteed if they are intended to use for valves. In other words, they can withstand the pressure of fluids without mechanical damages of the structures. Regarding this, we measured the compressive moduli at 25 and 50 °C (Supplementary Fig. S6), and the values were 1.8 × 10^4^ Pa at 25 °C and 1.3 × 10^5^ Pa at 50 °C. At 25 °C, the values were comparable with hydrogel structures formed by other types of hydrogel colloids^48^: shear modulus of 19,700 Pa. Second issue is the sustainability or long term stability of the structures in aqueous systems. We monitored the shapes of architectures for 6 months, and it was found that the hydrogel architectures still retain their original shapes. For some purposes, the stability of the architectures could be further improved by treating them with glutaldehyde to chemically connect between the hydrogel colloids. Then, the moduli were slightly increased to 4.8 × 10^4^ Pa at 25 °C and 2.1 × 10^5^ Pa at 50 °C, and the responses of the architectures became a little slower (93–107 s).

## Conclusions

We propose a new class of three-dimensional hydrogel valves and architectures implementing multiple functions (volume and color changes) in response to dual stimuli (temperature and ions). Unlike the hydrogels through the molecular crosslinking reactions, the present hydrogel architectures were made from the assembly of hydrogel and gold nanocolloids: sensing functions of the hydrogels were achieved by incorporation of hybrid colloids where the gold colloids were assembled on the surface of hydrogel colloids used as building blocks for the structures. The principle of operation of optical sensibility was differed from the previous approaches where photonic crystal structures should be retained in the hydrogel matrices^6^,^7^,^8^,^9^,^10^,^11^,^12^,^13^,^14^,^15^,^16^,^17^,^18^,^19^,^20^,^37^,^38^. Practically, they were rapidly manufactured with high moldability, and hence three-dimensional architectures with any shape can be easily produced. Moreover, the plasmonic on/off valve systems fabricated by using the present approach were effective in controlling flow rates of aqueous fluids by signalling liquid temperature and ionic conditions via changes in color, which might be helpful in various flow control systems where aqueous conditions (e.g., glucose or ion concentration changes) should be visually notified^21^. The present approach might be also helpful in developing novel biomimetic systems.

## Materials and Methods

### Materials

HAuCl_4_·3H_2_O (99.9%), *N,N′*-methylene bis-(acrylamide) (99%), allylamine (98%), potassium persulfate (>99%), sodium phosphate monobasic dihydrate (≥99.0%) sodium sulfate anhydrous (99%), sodium chloride (99.5%), *N,N,N′,N′*-tetramethylethylenediamine (99%), and ammonium persulfate (≥98.0%) were purchased from Sigma-Aldrich (Yongin, Korea). *N*-isopropylacrylamide (98%) was purchased from Wako Chemical Industries, Ltd. (Japan). Tri-sodium citrate (99%) was purchased from Fisher Scientific (USA). Sodium carbonate anhydrous (99.0%) was purchased from Junsei Chemical (Japan). Sylgard 184 was purchased from Dow Corning (USA). All chemical compounds were used as received without further purification.

### Synthesis of poly (*N*-isopropylacrylamide-co-allylamine) hydrogel colloids and Au NPs

For the preparation of the hydrogel architectures, 2.5 g *N*-isopropylacrylamide and 0.1 g *N,N′*-methylene bis(acrylamide) were dissolved in deionized water (100 mL) and equilibrated at 80 °C. Then, 81 μL allylamine and 2 mL potassium persulfate (0.031 g/mL) were consecutively added to the reactor. The mixture was stirred at 80 °C for 2–3 h and then quickly cooled in an ice bath and stored in a refrigerator before use.

For the synthesis of 15-nm (diameter) spherical Au NPs, 95 mL of 0.26 mM HAuCl_4_ aqueous solution was heated to reach an equilibrium at 100 °C for 40 min, and then 5 mL of tri-sodium citrate aqueous solution (0.5 wt%) was added to the HAuCl_4_ aqueous solution. After 30 min, the mixture was cooled to room temperature and stored at 4 °C. We referred to the methods listed in literatures for the synthesis of 33 and 50 nm spherical Au nanoparticles^45^,^46^.

### Preparation of hybrid colloids

The Au NPs were purified via centrifugation, the supernatant was removed, and the remaining Au NPs were re-dispersed in a tri-sodium citrate aqueous solution (0.85, 0.88 and 0.92 mM tri-sodium citrate aqueous solution for 15, 33, and 50 nm, respectively). Poly(*N*-isopropylacrylamide-co-allylamine) hydrogel colloids were also centrifuged and re-dispersed in deionized water. The hybrid colloids were prepared via the addition of precise quantities of hydrogel colloid aqueous dispersions to the Au NP aqueous dispersion^46^. The mixture was then slightly shaken and equilibrated at 25 °C for 30 min, followed by heating to 50 °C for 30 min and cooling to 25 °C. UV-vis spectroscopy (Cary 50, Agilent, USA) was used to obtain UV-vis spectra of the hybrid colloids aqueous dispersions.

In order to investigate the temperature-dependent optical characteristics, the hybrid colloids at 25 °C were consecutively equilibrated by heating to 32, 37, and finally 50 °C. The equilibrium time at each temperature was 30 min. After the equilibrium, the colors and UV-vis spectra of the aqueous dispersion were recorded. Scanning electron microscopy (SEM, S-4800, Hitachi, Japan) was also used to investigate the temperature-dependent morphologies of the hybrid colloids. 10 μL of hybrid colloid aqueous dispersion equilibrated at either 25 °C or 50 °C for 1 h was dropped onto a silicon wafer (equilibrated at each temperature for 5 h). SEM images of the hybrid particles were acquired without sputtering Au or Pt onto the samples.

### Fabrication and characterization of cylindrical plasmonic hydrogel architectures

The hybrid and hydrogel colloids were mixed in 5-mL cylindrical glass vials with appropriate particle ratios depending on the type of Au NPs. The mixture was then equilibrated at 60 °C for 30 min. The clear aqueous solution was removed using a glass pipette, followed by the addition of the same volume of deionized water equilibrated at 25 °C. After equilibrating the hydrogel architectures at 60 °C, the purification process was repeated an additional time. The compressive moduli (Supplementary Fig. S6) of the hydrogel architectures were measured with a rheometer (ARES, TA Instruments, USA). In these experiments, the hydrogel architectures were first equilibrated in water at 25 and 50 °C for 24 h and uniaxial compression experiments were subsequently performed on the hydrogel architectures in water at 25 and 50 °C, respectively.

For measurement of the response times (relaxation times) of the hydrogel architectures (Supplementary Fig. S5), the hydrogels were first equilibrated at 25 °C for 24 h and subsequently transferred to a water bath at 50 °C (for heating) or the hydrogels equilibrated at 50 °C for 24 h were subsequently transferred to a water bath at 25 °C (for cooling). The dimensional changes of the hydrogel architectures with time were measured by analysing movies. To obtain the response time of bulk gels, we fabricated the bulk hydrogels with the same chemical composition as the hydrogel colloids. For the preparation of the bulk gels, 0.2 wt% ammonium persulfate and 0.3 vol% *N,N,N′,N′*-tetramethylethylenediamine were added to a 15 mL of mixture (*N*-isopropylacrylamide, allylamine and *N,N′*-methylene bis(acrylamide)). After the reaction at room temperature for 24 h, the bulk hydrogels were washed with deionized water. The response time of bulk hydrogels was also measured by using the same process as described above.

### Fabrication of plasmonic hydrogel fish and butterfly

Temporary polypropylene molds in fish and butterfly shapes were formed and placed on a Petri dish. PDMS precursor solution was poured into the space between the Petri dish and polypropylene mold, and the PDMS solution was fully cured at room temperature for 24 h. After removing the temporary mold, the aqueous dispersion containing the hydrogel colloids and hybrid colloids (containing 33 nm Au NPs) were poured into the PDMS mold, and the dispersion was heated to 60 °C for 30 min. The washing and purification step was as described above.

### Temperature- and ion-dependent volume and color changes of plasmonic hydrogel architectures

For temperature-dependent studies, the three cylindrical hydrogel architectures at 25 °C were sequentially equilibrated at 32, 35, 37, 50 and 60 °C for 30 min. At each equilibrium temperature, the shapes and colors of the architectures were photographed. To investigate the reversibility, they were repeatedly heated to 60 °C and cooled to 25 °C three times, and shape and colors of the hydrogel architectures were also recorded. For ion-dependent studies, various sodium salts (Na_3_citrate, Na_2_SO_4_, NaH_2_PO_4_, and NaCl) dissolved in deionized water were added to cylindrical hydrogel architectures (containing 33-nm Au NPs) at 25 °C. After 30 min, the shapes and colors of the architectures were recorded in a photograph. The salt-containing aqueous solutions were then removed from each well, followed by the addition of deionized water. After 30 min, the shapes and colors of the architectures were photographed.

For temperature-dependent studies with the plasmonic hydrogel, molds in both fish and butterfly shapes (containing 33-nm Au NPs) were sequentially equilibrated at 25, 32, 50, and 25 °C for 30 min. The shapes and colors of the architectures were recorded via a photograph. Likewise, the ion-dependent studies of the architectures were conducted by adding Na_3_citrate (0.15 and 0.3 M) aqueous solutions to the architectures. Reversibility was observed by replacing the salt-containing aqueous solution with deionized water. At each step, the shapes and colors of the architectures were recorded photographed.

### Fabrication and flow rate “on-off” tests of plasmonic hydrogel valves

The plasmonic valve (with 33-nm Au NPs) fabricated in a cylindrical glass vial was inserted into a glass tube (i.d. 1 cm). In a tube, either deionized water or salt-containing aqueous solution was added from a syringe pump at a fixed flow rate (0.2 mL/min). The tube outlet was capped with a PDMS block and an 18-G needle was inserted to allow for water or the aqueous solution to be released through the needle. The temperature of the flow tube was modulated from a water circulating tube surrounding the outside of the flow tube. The flow rate of aqueous solution going out through the needle was checked every 30 s by measuring the cumulative weight of the aqueous solution leaving the flow system. Temperature-dependent “on” and “off” tests of the plasmonic valves were performed with either repeated heating (50 °C) or cooling (25 °C) of the flow tube every 15 min. During the test, the cumulative volume of deionized water was measured, and the colors of the valve were recorded via video. Salt-dependent “on” and “off” tests of valves were performed by repeating the influx of Na_2_CO_3_ aqueous solution (0.5 M) and deionized water every 15 min.

## Additional Information

**How to cite this article**: Song, J. E. and Cho, E. C. Dual-responsive and Multi-functional Plasmonic Hydrogel Valves and Biomimetic Architectures Formed with Hydrogel and Gold Nanocolloids. *Sci. Rep*. **6**, 34622; doi: 10.1038/srep34622 (2016).

## Supplementary Material

Supplementary Information
