# Supplementary material for: Dual-responsive and Multi-functional Plasmonic Hydrogel Valves and Biomimetic Architectures Formed with Hydrogel and Gold Nanocolloids
Source: Sci Rep. 2016 Oct 5;6:34622. doi: 10.1038/srep34622 (PMC5050413; doi:10.1038/srep34622)
Supplement: Supplementary Information [file srep34622-s1.doc]

Supplementary Information

Dual-responsive and Multi-functional Plasmonic Hydrogel Valves and Biomimetic Architectures Formed with Hydrogel and Gold Nanocolloids

Ji Eun Song & Eun Chul Cho*

Department of Chemical Engineering, Hanyang University, Seoul, 04763, South Korea. Correspondence and requests for materials should be addressed to E. C. C. (email: enjoe@hanyang.ac.kr)


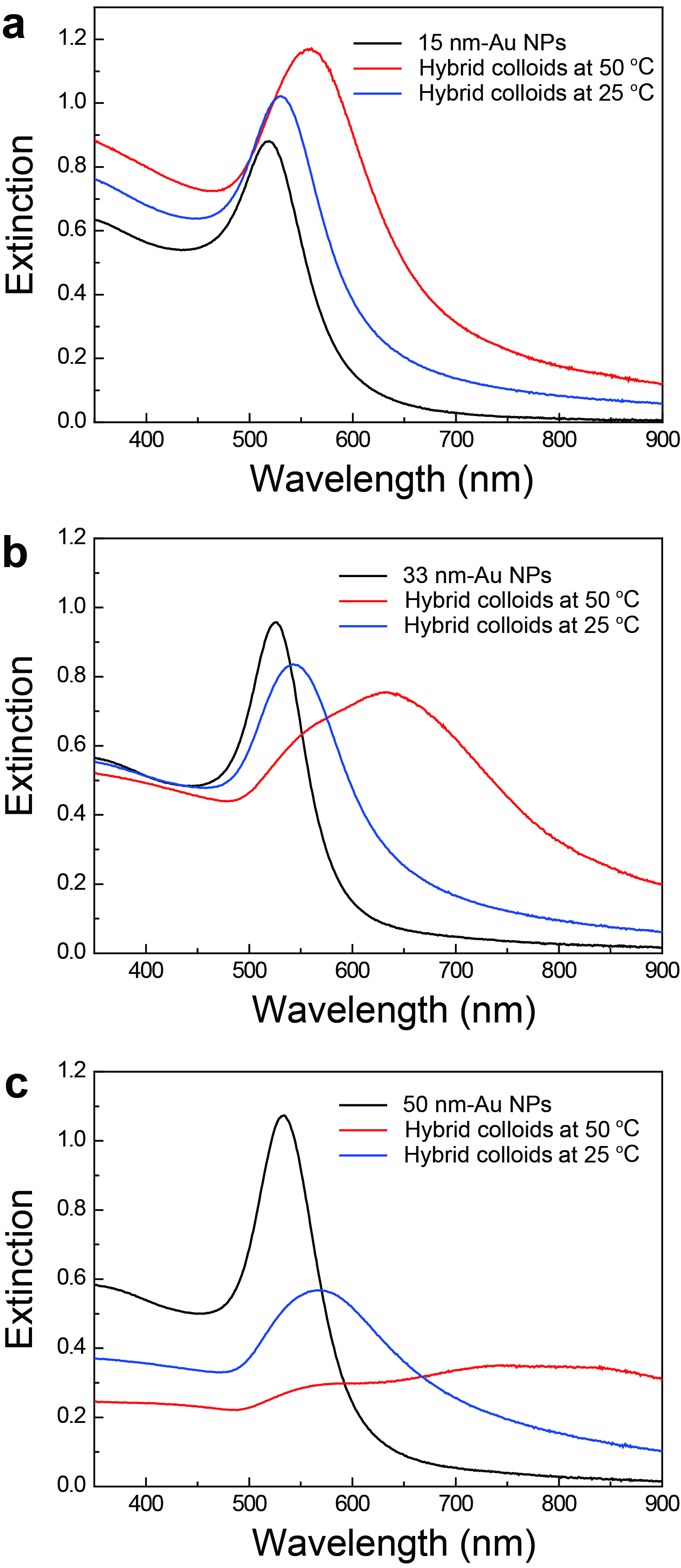


**Supplementary Fig. S1.** **UV-vis spectra of Au NPs aqueous dispersion and hybrid colloids formed with Au NPs and hydrogel colloids.** The diameters of Au NPs used were (**a**) 15 nm, (**b**) 33 nm, and (**c**) 50 nm. The temperature-dependent changes in spectral bandwidth of the hybrid colloids were increased with increasing the Au NPs sizes used.


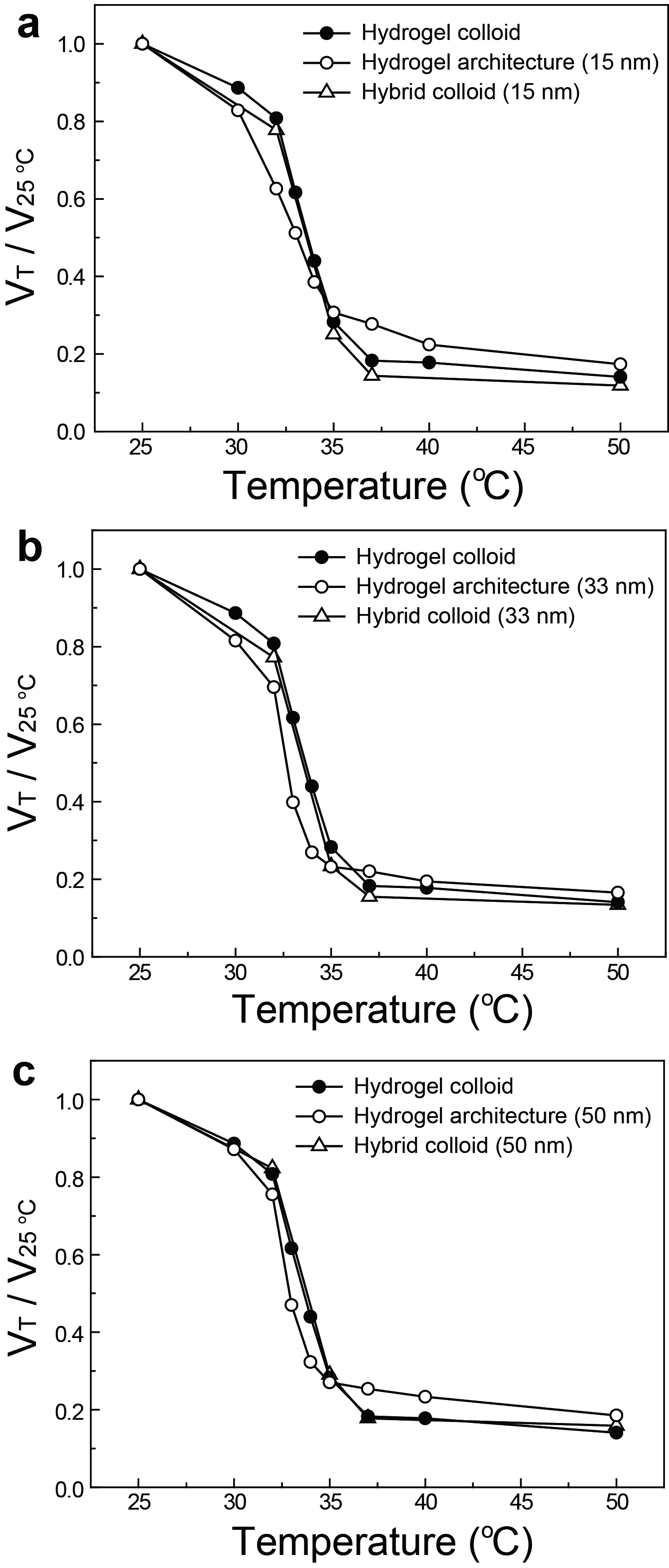


**Supplementary Fig. S2.** **Temperature-dependent changes in volume of hydrogel colloids, hydrogel architectures (containing hybrid colloids), and hybrid colloids.** The sizes of Au NPs used for the hydrogel architecture and the hybrid colloid were (**a**) 15 nm, (**b**) 33 nm, and (**c**) 50 nm. The temperature-dependent volume changes of the hydrogel and the three hybrid colloids were determined from the measurement of hydrodynamic diameters. The temperature-dependent volume changes of the three hydrogel architectures were determined by analyzing photographs of the hydrogel architecture. In each Au size used, the volumetric changes of the hydrogel colloid and the three hybrid colloids were nearly same.


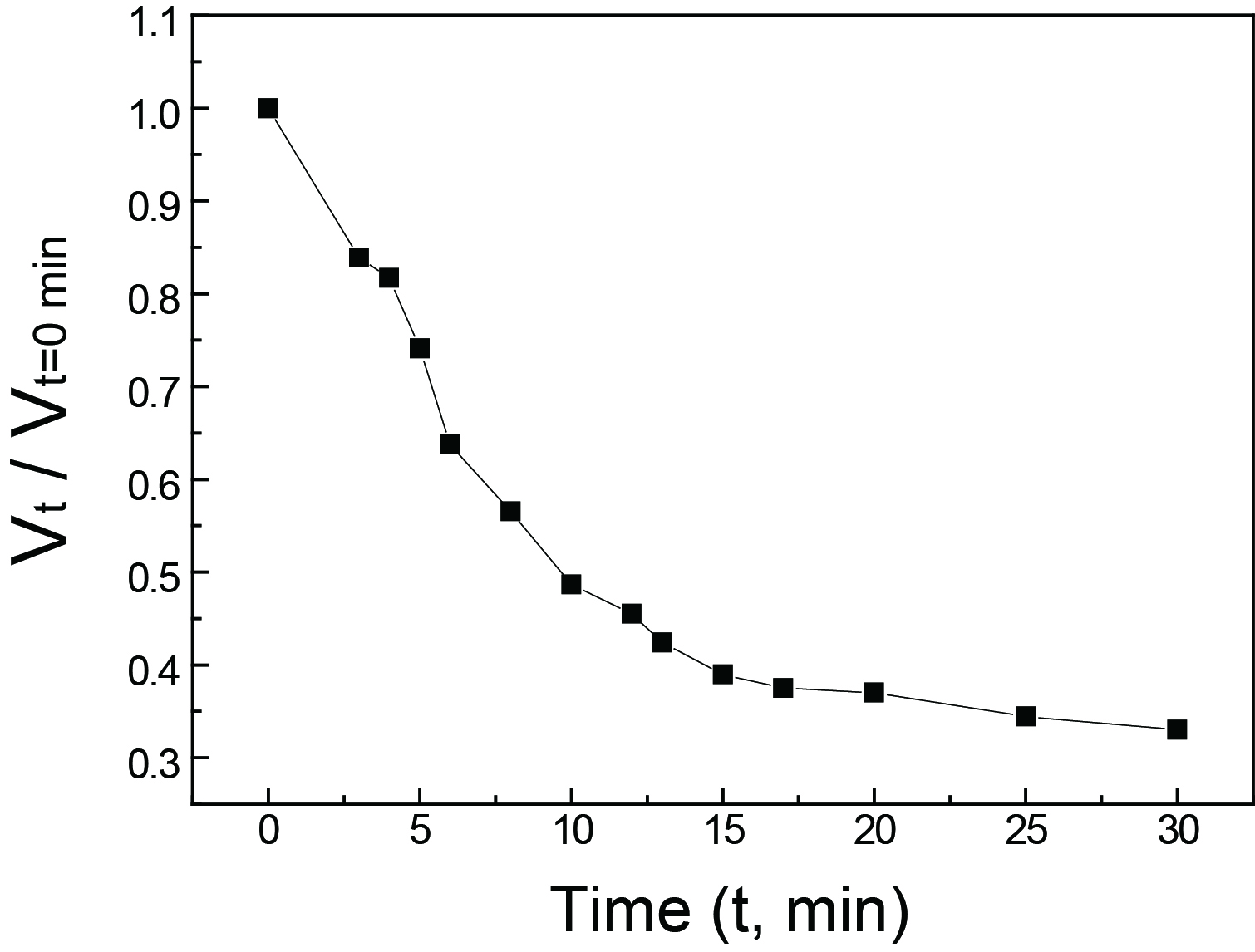


**Supplementary Fig. S3.** Time-course volumetric changes of an aqueous dispersion of hydrogel colloids and hybrid colloids (containing 50 nm Au NPs) in a cylindrical glass vial during the period of heating at 60 ^o^C. The aqueous dispersion rapidly formed a plasmonic hydrogel architecture having a cylindrical shape within 30 min.


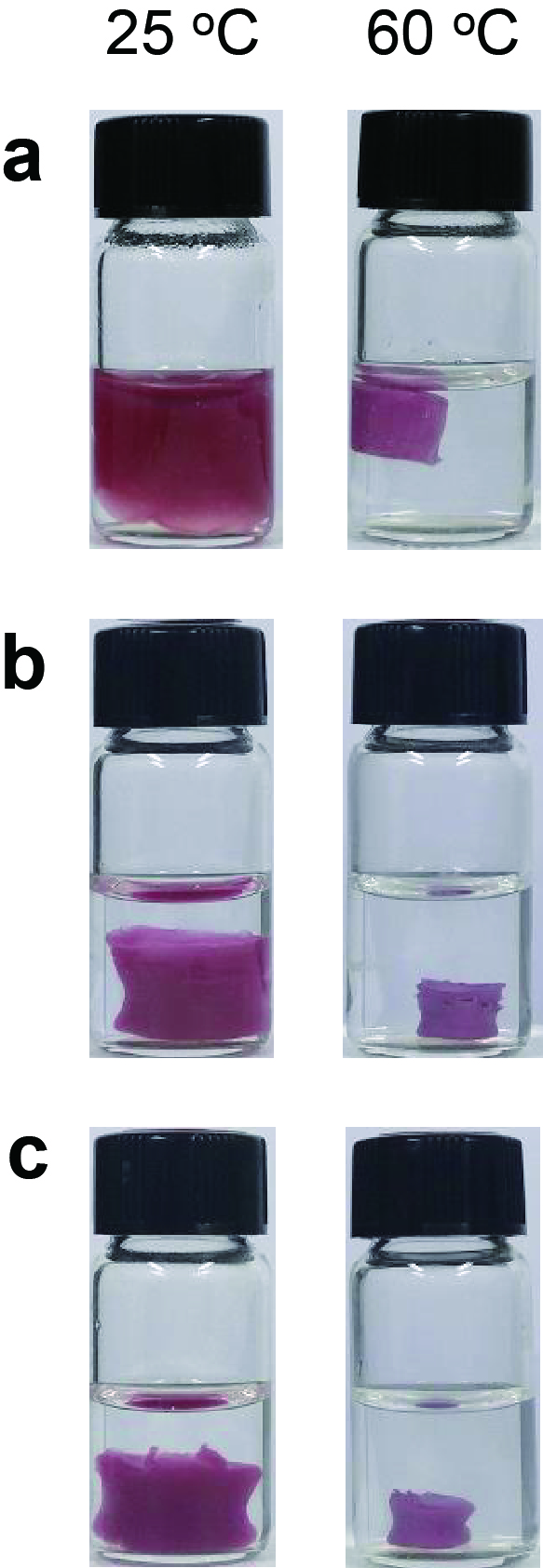


**Supplementary Fig. S4.** **Photographs displaying temperature-dependent changes in shapes and colors of colloidal hydrogel architectures containing Au NPs (not hybrid colloids).** The architectures were made from heating the aqueous dispersion containing hydrogel colloids and Au NPs with (**a**) 15 nm, (**b**) 33 nm, and (**c**) 50 nm. Note that we did not use hybrid colloids. Contrast to color changes in plasmonic hydrogel architectures (containing hybrid colloids), the hydrogel architectures consisting of Au NPs and hydrogel colloids did not show any appreciable color change.


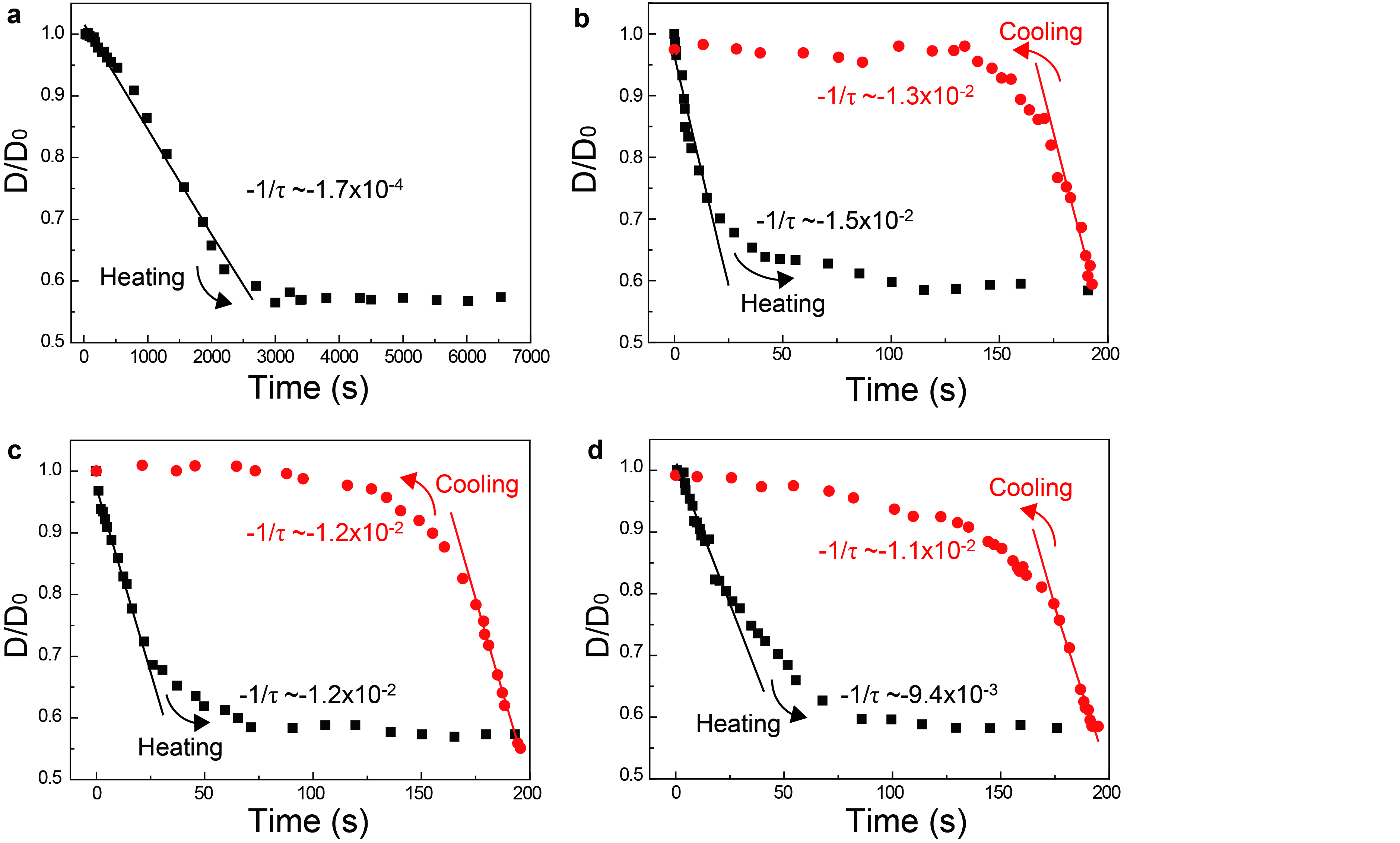


**Supplementary Fig. S5.** Time-dependent evolution of dimensional changes, *D(t)/D_0_*, of (**a**) a bulk hydrogel, (**b**) colloidal hydrogel architectures containing hybrid colloids (with 15 nm gold nanoparticles), (**c**) colloidal hydrogel architectures without hybrid colloids, and (**d**) colloidal hydrogel architectures (without hybrid colloids) which are treated with glutaldehyde after the formation of hydrogel architectures. *D*_0_ is the initial size. From the exponential fits in the plots, we obtained the response (relaxation) times (τ) of four types of hydrogels. In these measurements, the hydrogels were first equilibrated at 25 ^o^C for 24 h and subsequently transferred to a water bath at 50 (heating) or the hydrogels equilibrated at 50 ^o^C for 24 h were subsequently transferred to a water bath at 25 ^o^C (cooling). For the glutaldehyde-treated hydrogel architectures, approximately 1 vol% glutaraldehyde aqueous solution was introduced to the hydrogel architectures (without Au NPs). The reaction was performed at 65 ^o^C for 1 h, followed by repeatedly washing the hydrogel architectures with deionized water.

**
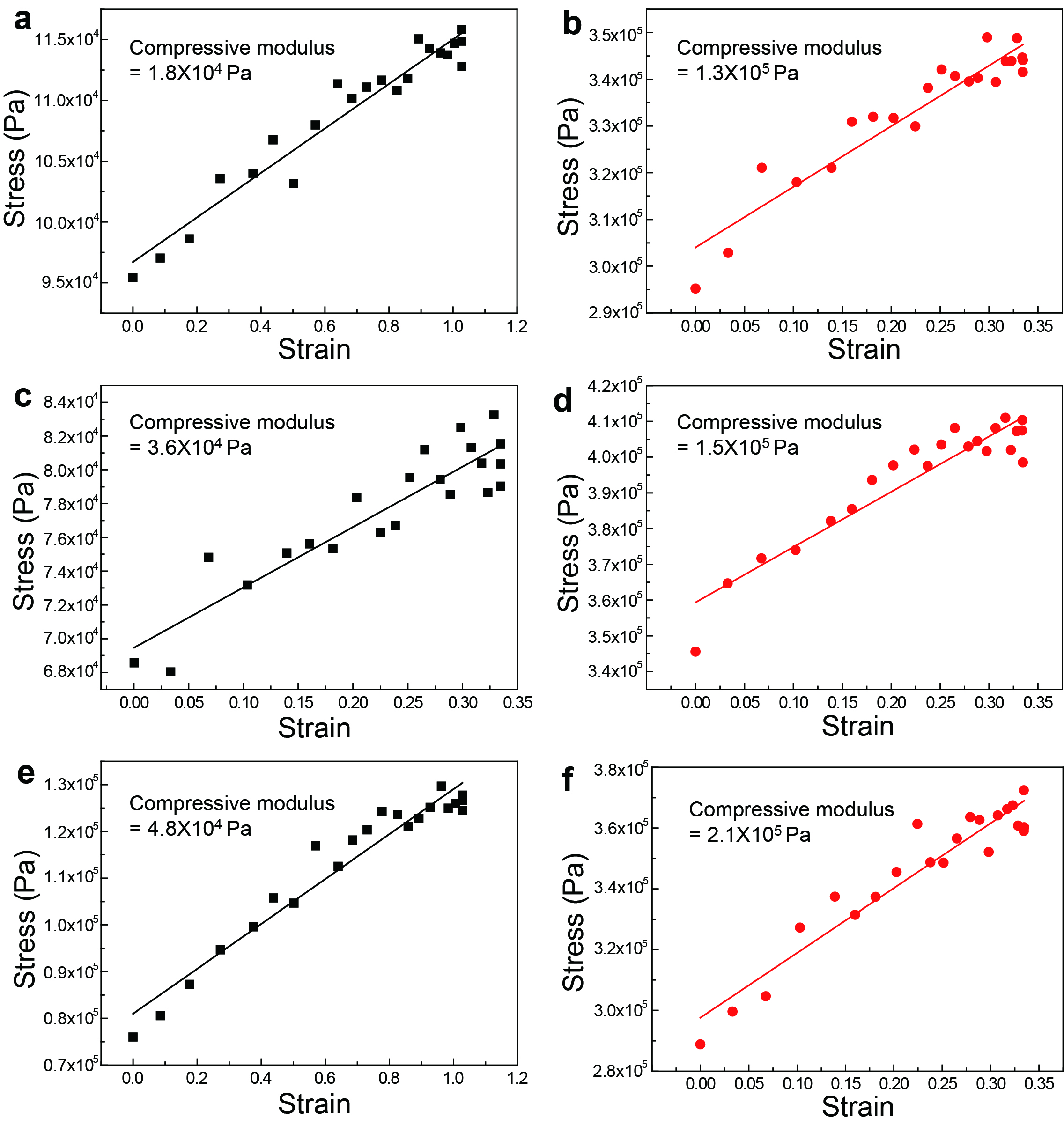
**

**Supplementary Fig. S6.** Compressive stresses versus strains of (**a**,**b**) colloidal plasmonic hydrogel architectures containing hybrid colloids (bearing 15 nm-sized Au NPs), (**c**,**d**) colloidal hydrogel architectures without hybrid colloids, and (**e**,**f**) the hydrogel architectures after the treatment of glutaldehyde. The measurement are conducted at (**a**,**c**,**e**) 25 ^o^C and (**b**,**d**,**f**) 50 ^o^C.
